# Associations among soil microbial and plant richness across global terrestrial biomes

**DOI:** 10.1093/ismeco/ycag266

**Published:** 2026-09-18

**Authors:** Samuel Bickel, Ahmed Abdelfattah, Ayco J M Tack, Wisnu Adi Wicaksono, Gabriele Berg

**Affiliations:** Institute of Environmental Biotechnology, Graz University of Technology, 8010 8010 Graz, Austria; Microbiome Biotechnology, Leibniz Institute for Agricultural Engineering and Bioeconomy (ATB), 14469 14469 Potsdam, Germany; Department of Ecology, Environment and Plant Sciences, Stockholm University, SE-106 91 Stockholm, Sweden; Institute of Environmental Biotechnology, Graz University of Technology, 8010 8010 Graz, Austria; Institute of Environmental Biotechnology, Graz University of Technology, 8010 8010 Graz, Austria; Microbiome Biotechnology, Leibniz Institute for Agricultural Engineering and Bioeconomy (ATB), 14469 14469 Potsdam, Germany; Institute for Biochemistry and Biology, University of Potsdam, 14469 Potsdam, Germany

**Keywords:** soil microbial richness, fungal endophyte diversity, plant-microbiome interactions, biogeographical distribution, environmental drivers of richness, cross-kingdom associations

## Abstract

Soil microbiomes play a crucial role in terrestrial ecosystems by influencing plant diversity and ecosystem functions, yet global patterns linking soil microbial and plant species richness remain unclear. Here we employed machine learning models integrating environmental variables to map global richness patterns of plants, bacteria, and fungi across terrestrial biomes and examined their associations. We found distinct biogeographical distributions and high diversity regions: plant richness peaked in tropical forests, soil bacterial richness was highest toward arid and transitional zones, and soil fungal richness was prominent in tundra and boreal forests. Notably, plant richness correlated positively with microbial richness and strongly with fungal endophyte richness, supporting the concept that diversity fosters further diversity. These findings reveal complex cross-kingdom interactions shaping terrestrial biodiversity and underscore the importance of integrated conservation strategies that consider multiple organismal groups and their environmental drivers.

## Introduction

Plants and their associated microorganisms have co-evolved through sustained cross-kingdom interactions that have reciprocally shaped their evolutionary trajectories. Soil acts as a complex, porous habitat that maintains vast microbial genetic diversity, serving as the reservoir from which plant microbiomes are assembled. Interactions among bacteria, fungi, and plants, within and across kingdoms, are essential for plant survival and health [1, 2]. Because most plants depend on soil, the soil microbiome significantly influences plant fitness [2, 3] and constitutes a substantial portion of terrestrial biomass, together with plants, bacteria, and fungi [4]. Microbial interactions in soil are governed by environmental factors such as soil properties, climate, and biological processes that vary across biomes [5, 6]. Community assembly is further shaped by host selection pressures, whereby plant roots and other organisms filter microbial taxa along the soil-root continuum [7], and by stochastic processes, including ecological drift and dispersal, whose relative importance changes with environmental conditions such as soil pH [8]. These cross-kingdom interactions and the independent responses of plants and microbes to environmental drivers fundamentally shape the biogeographical patterns of bacteria, fungi, and plants across terrestrial ecosystems.

Microbial diversity in terrestrial ecosystems is intricately connected to plant diversity. Plants release root exudates that shape the soil microbiome, while the soil microbiota supports essential ecosystem functions [9, 10], including soil formation, nutrient cycling, mineralization, water retention, recycling of organic residues, detoxification, and disease suppression [11–16]. Despite these strong mutual dependencies that have evolved and diversified together over millions of years, the associations between plant and microbial diversity remain complex and context dependent. For example, grasslands exhibit stronger correlations between plant and fungal diversity than with bacterial diversity, although both fungal and bacterial diversity are influenced by plant traits [12]. Environmental factors, such as rainfall frequency, soil pH, and carbon inputs via primary productivity, critically mediate these cross-kingdom associations [11, 12, 17] often confounding observed patterns. Moreover, the drivers of diversity can vary among fungal functional guilds, with fungi exhibiting divergent biogeographical patterns that reflect their distinct life strategies and interactions with both plants and bacteria [17–19]. The complexity arising from intertwined plant-microbe and microbe-microbe interactions across biomes complicates efforts to generalize soil–plant diversity associations. To date, no comprehensive analysis has integrated the biogeographical diversity patterns of plants, bacteria, and fungi at the global scale to identify macroecological trends and potential cross-kingdom diversity co-variation.

Two principal hypotheses have historically been proposed to elucidate how within-kingdom (e.g. bacteria-bacteria) and between-kingdom (e.g. plant-microbe) interactions can shape macroecological diversity patterns. The “ecological controls” (EC) hypothesis [20, 21] suggests that the evolution or migration of new species becomes limited as existing niches are filled due to resource limitation, thereby constraining diversity [21]. Conversely, the “diversity begets diversity” (DBD) hypothesis [22, 23] contends that an increase in diversity fosters further diversity through mechanisms such as niche construction and interspecific interactions. Research has provided insights into these dynamics, demonstrating that DBD is particularly robust in low-diversity microbiomes [24]. In these environments, initial species interactions may promote the establishment of new taxa, thus enhancing overall diversity. However, in high-diversity ecosystems, the DBD effect weakens, aligning with EC predictions that, as niches reach saturation, diversity stabilizes and stops accumulating further [24, 25]. This nuanced interplay suggests that while diverse plant communities can catalyze microbial diversity through varied root exudates and niche provisioning, ecological limitations, such as competitive exclusion and resource depletion, impose a ceiling on cross-kingdom diversity accumulation as ecosystems mature. Furthermore, experimental studies have indicated that biodiversity can create conditions that facilitate novel speciation through positive feedback mechanisms, particularly where resources become limited due to high diversity [23, 26]. This framework thus emphasizes that the relationship between ecological interactions and microbiome diversity is intricate, requiring a contextual understanding of microbial diversity and its associations with plant diversity at the biome scale.

In this study, we focus on the diversity of plants, bacteria, and fungi, which together comprise the primary constituents of terrestrial biomass [4]. Our objective is to map global richness (number of taxa) of the three kingdoms and examine whether soil bacterial and fungal richness, or the richness of specific fungal guilds, can explain plant richness at the global scale. Specifically, we aim to (i) identify the global biogeography of plant, bacterial, and fungal richness and their common environmental drivers, and (ii) test the associations between soil fungal and bacterial richness and plant species richness using predicted global maps (see Fig. 1).

**Figure 1:**
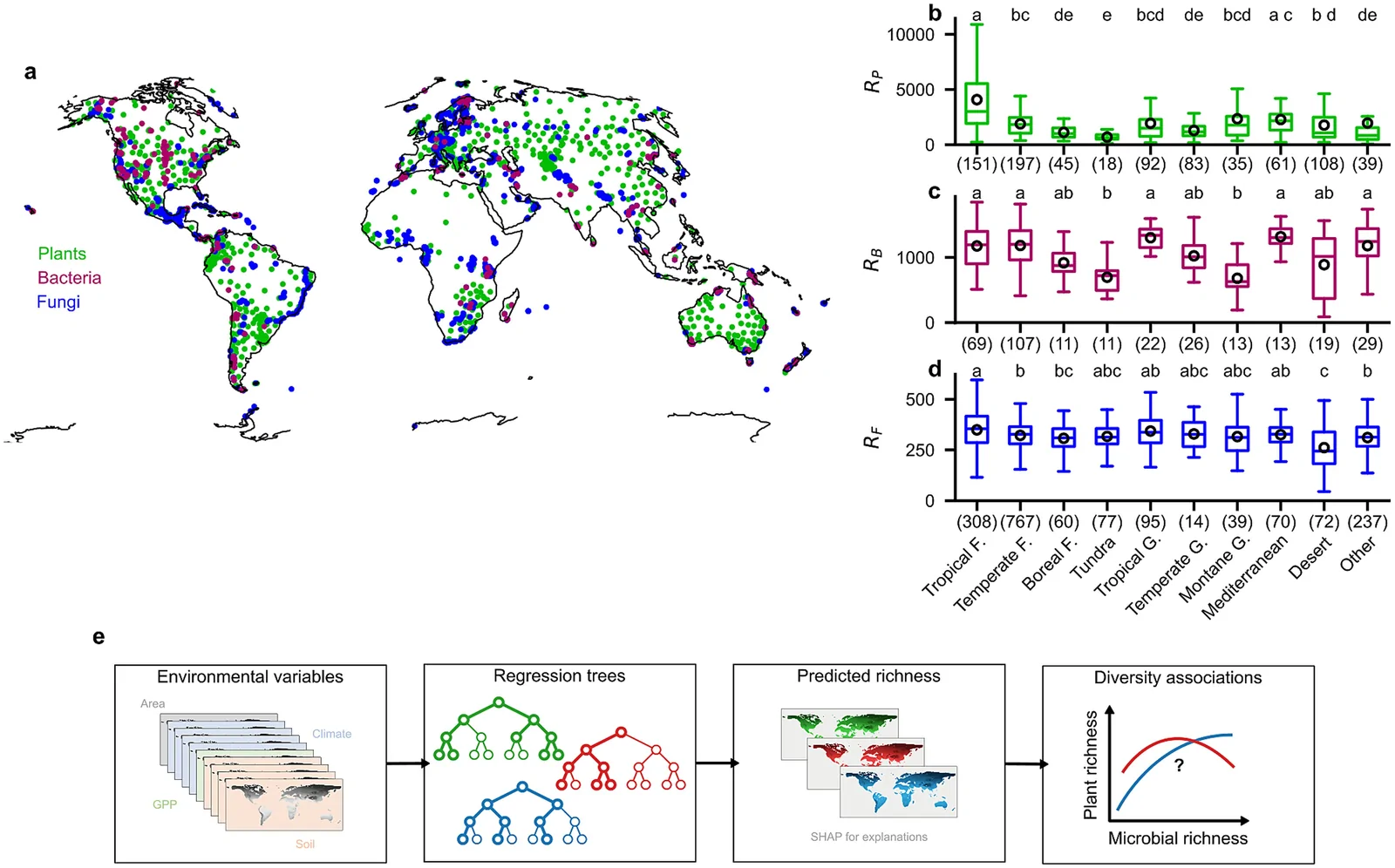
Global mapping for plant and soil microbial richness associations. (a) Spatial distribution of sampling locations for plant, bacterial, and fungal richness obtained from previously published datasets [27–29]. (b–d) Observed richness of plants (*R_P_*), bacteria (*R_B_*), and fungi (*R_F_*). The numbers in brackets indicate the number of samples. Letters indicate significant differences at α = 0.05 based on Dunn’s test with Bonferroni correction. (e) Ten environmental variables were used to train regression trees and predict biogeographical patterns. The trained models are used to investigate links between plant and microbial richness.

## Materials and methods

### Global mapping of soil microbial richness and plant richness

We compiled three distinct datasets to model the global biogeography of plant, bacterial, and fungal richness. Using gradient boosting and random forest regression, we generated global richness maps. For model training, global datasets on community diversity for bacteria [27], fungi [28], and vascular plants [29] were used. The fungal data were subsampled to uniform sequencing depths (*n* = 1003) and the other two datasets were used as provided. All point observations were aggregated to a common spatial scale of 0.1 degrees using the sample means, and these “sample locations” were used for further modeling. This resulted in the following number of sample locations (percentiles *P_%_* and maximum number of aggregated samples): 829 for plants (*P*_25_ = 1, *P*_50_ = 1, *P*_75_ = 1, max. = 2), 320 for bacteria (*P*_25_ = 1, *P*_50_ = 1, *P*_75_ = 2, max. = 39), and 1739 for fungi (*P*_25_ = 1, *P*_50_ = 1, *P*_75_ = 2, max. = 69). To account for variability between datasets (taxonomy, sequencing, etc.), we used standardized richness values (*z*-score) for our analysis. Biomes were grouped following the Olson classification [30]. The environmental variables were added based on the coordinates of the sample locations (latitude and longitude) and were obtained from a variety of sources. Data with global coverage were used, and all raster layers were re-gridded to 0.1 degrees by averaging. Soil data were obtained from SoilGrids [31]. A variable “soil type diversity” was introduced as a measure of sub-grid heterogeneity by counting the number of World Reference Base (2006) classes at 0.1-degree resolution (aggregating SoilGrids [31] information at 250 m resolution). Mean annual temperature and mean solar radiation were obtained from WorldClim [32]. Mean annual precipitation and the mean rainfall frequency were calculated [33] from MSWEP [34]. Mean annual gross primary productivity was obtained from GOSIF [35]. All variables and their units are summarized in Table S1.

We stacked random forest and gradient boosting trees (XGBoost [36]) to train three biogeographical distribution models for plant, bacterial, and fungal richness using ten environmental variables (Table S1). The pipeline for model fitting, hyperparameter tuning, and model averaging was implemented with sklearn [37] including the preprocessing and model selection. The calibrated stacked random forests and boosting trees were averaged before applying linear bias correction [38, 39]. To evaluate model performance, the data were split by withholding 25% of the data for testing. Using the remaining training data, we optimized the model hyperparameters using optuna [40] with 5-fold cross-validation by minimizing the root mean squared error. We performed model selection with hyperparameter tuning with different test sets (50 times) to address model variability. The best model was selected based on *R*^2^ and subsequently used for spatial predictions. We used multioutput random forest to simultaneously model the richness of the different fungal guilds [28] (regrouped from the original dataset to saprotrophs, endophytes, mycorrhizal fungi, parasites/pathogens, and mycoparasites/pathothrophs; Table S2). Otherwise, the pipeline for mapping the richness of fungal guilds was the same as described above.

### Determination of soil microbial diversity and associations with plant richness

Based on spatial maps of plant, bacterial, and fungal richness, we investigated the diversity associations considering three types of associations linking the richness of plants, bacteria, and fungi: no-association, linear, and curvilinear. To assess the soil diversity association, we fitted linear models with polynomial terms of degree zero, one, and two, omitting higher-order terms if the *R*^2^ did not improve by >1%. We derived both global and biome-specific associations to identify general trends and biogeographical patterns. To further analyze the impact of individual environmental drivers on the richness of plants, bacteria, and fungi, we used Shapley additive explanations [41] (SHAP), randomly selecting ten samples from each biome and scaling the SHAP values by the largest modeled richness value for each kingdom. We used the predicted richness of bacteria, fungi, and fungal guilds to model plant richness following the same modeling approach as described above (see section Global mapping of soil microbial richness and plant richness). Using the plant dataset, we extracted the richness values of bacteria, fungi, and the fungal guilds from our maps based on coordinates of the sample locations. Two models were constructed to predict plant richness: one using only bacterial and fungal richness, and another using bacterial richness and individual fungal guild’s richness. Feature importance was ranked based on the mean of absolute SHAP values to identify the most influential microbial group for explaining plant richness.

## Results

### Diversity patterns and their environmental drivers across biomes

Fungal richness exhibited less variation across biomes compared to bacterial and plant richness, as shown in both the observed data and the modeled global maps (Figs 1 and 2). The predictive models performed best for plant richness (*R*^2^ = 60 ± 7%, max. *R*^2^ = 76%, *n* = 829; 25% testing data), while performance was lower for bacterial richness (*R*^2^ = 41 ± 9%, max. *R*^2^ = 62%, *n* = 320) and lowest for fungal richness (*R*^2^ = 13 ± 6%, max. *R*^2^ = 22%, *n* = 1739) (Fig. S1). The spatial distribution of model uncertainty for plant and bacterial richness highlights undersampled regions (e.g. Sahara) while the uncertainty for fungal richness was comparatively low and spatially uniform (Fig. S2). To improve the fungal richness maps, we partitioned fungal richness into functional guilds using a multioutput random forest approach, which increased the explained variance across all guilds (*R*^2^ = 30 ± 2%, max. *R*^2^ = 34%, *n* = 1739) (Fig. S1). Globally, bacterial richness was highest in climatic transition zones such as the Sahel and tropical savannas, below average in the Amazon, and lowest in deserts (Fig. 2). Fungal richness was slightly elevated in central Africa and toward northern latitudes, whereas plant richness peaked near the equator, particularly in the Amazon, Central Africa, and Southeast Asia (Fig. 2a). While bacterial richness displayed clear biogeographical patterns, fungal richness lacked distinct patterns but was consistently low in deserts (Fig. 2b and c). These contrasting biogeographical patterns are evident, for example, between plants and bacteria in North America and in the Amazon basin (Fig. 2d).

**Figure 2:**
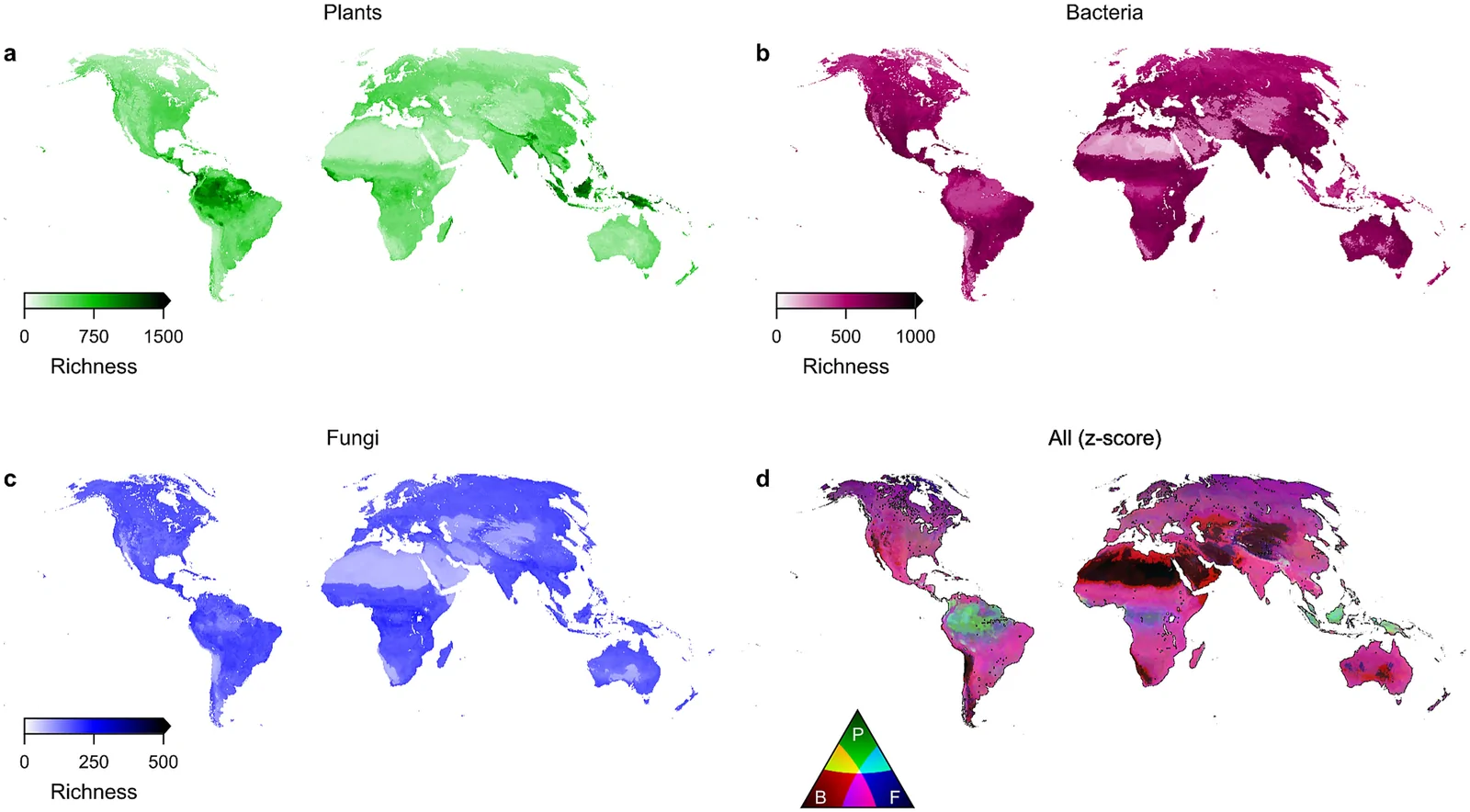
Global predictions of plant and microbiome richness. (a–c) Richness of plants, bacteria, and fungi. (d) Composite map based on standardized richness (*z*-score). The triangle shows the relative changes in richness with the center indicating the highest and equal richness of plants (P), bacteria (B), and fungi (F), respectively.

Despite some overlap, no single environmental variable emerged as a universal driver of richness across the three kingdoms. The selected environmental factors affected bacterial, fungal, and plant richness to different extents. High mean annual precipitation strongly increased plant and fungal richness; the latter was strongly reduced at low mean annual precipitation (Fig. 3a and b). Gross primary productivity was the only consistent predictor for increased microbial and plant richness (Fig. 3a and c). Although mean annual temperature influenced all groups similarly, it did not appear as a major determinant of richness. In contrast, rainfall frequency strongly influenced plant and bacterial richness but had no discernible effect on fungal richness (Fig. 3d). Soil edaphic factors, particularly soil pH (Fig. 3e) and bulk density, shaped bacterial richness, while soil texture had only a minor impact. Interestingly, high soil type diversity strongly reduced fungal richness, although it had a small positive impact on bacterial and plant richness (Fig. 3a). The spatial distributions of important environmental drivers with diverging patterns among plant, bacterial, and fungal richness (Fig. 3b–e) are displayed in Fig. S3.

**Figure 3:**
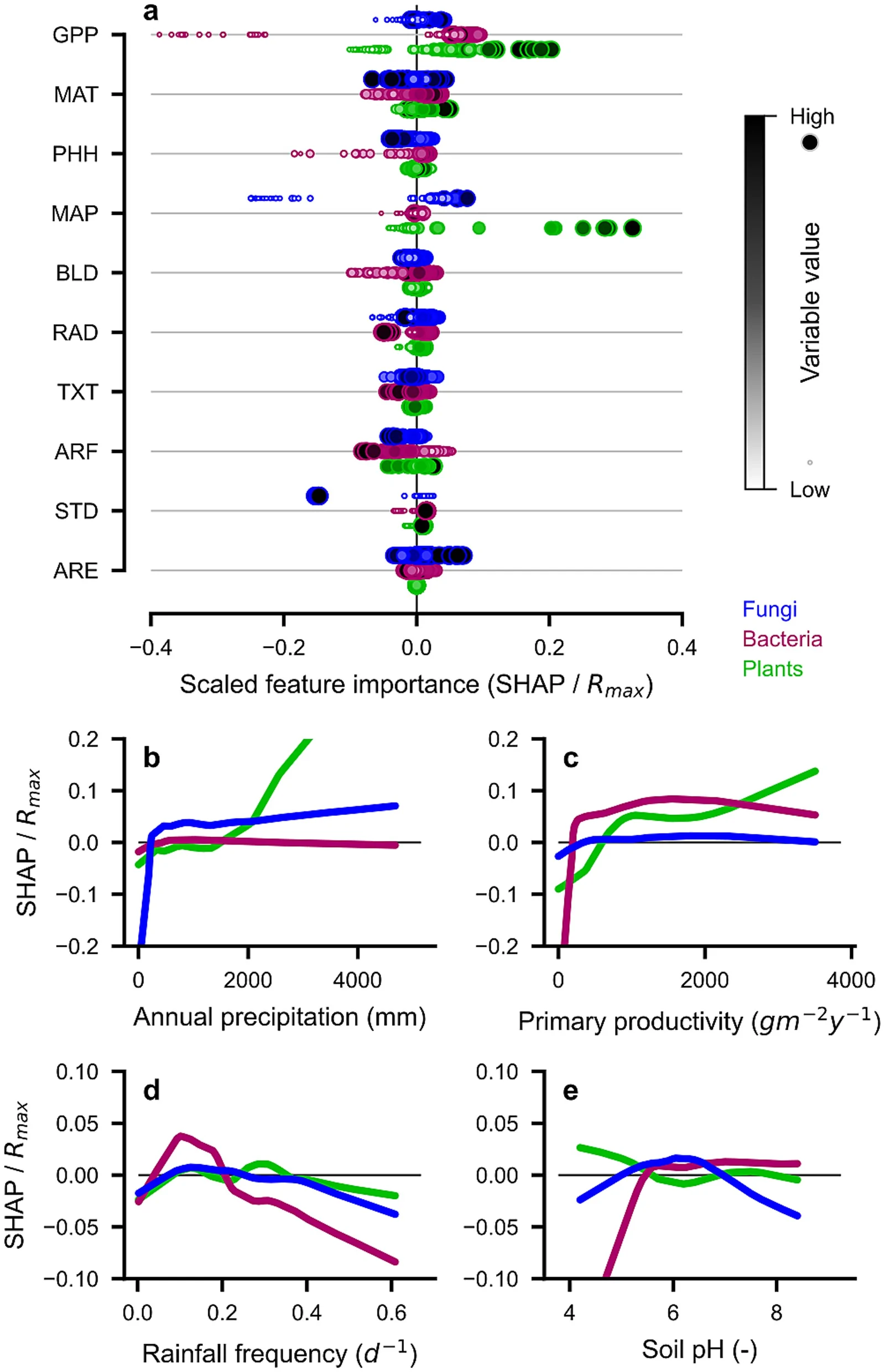
Environmental drivers of plant and soil microbiome richness. (a) Scaled feature importance for explaining shifts in mean richness. The variables are: area of gross primary productivity (GPP), mean annual temperature (MAT), soil pH in water (PHH), mean annual precipitation (MAP), bulk density (BLD), radiation (RAD), soil texture (TXT), average rainfall frequency (ARF), soil type diversity (STD), and sampling grid cell (ARE). The Shapley additive explanations [41] (SHAP) values were divided by the maximum richness observed for each kingdom (*R_max_*). Darker shading indicates higher values of the environmental variable. (b–e) Smoothed SHAP values with respect to MAP, GPP, ARF, and PHH.

### Cross-kingdom associations: how fungal guild richness links to plant diversity

On average, tropical forests exhibited the highest plant richness but relatively low microbial richness. Bacterial richness was high in tropical grassland and Mediterranean regions, where both plant and fungal richness were relatively low. Fungal richness showed less variation across biomes, except for being much lower in deserts in agreement with previous studies [42, 43]. Notably, both bacterial and plant richness were low in boreal forests and tundra, where fungal richness remained relatively high (Fig. 4a). In most biomes, modeled plant richness was positively correlated with microbial richness; however, this correlation was strongly negative for bacterial richness in tropical forests and weakly negative for fungal richness in tropical forests, boreal forests, and tundra (Fig. 4b). When pairing the sampling data at spatial scales above 0.1°, bacteria–fungi and fungi–plant correlations were positive regardless of scale while bacteria–plant correlations appeared positive only when aggerating to scales larger than 10° (Fig. S4). Overall, modeled plant richness increased linearly with fungal richness, whereas bacterial richness showed a saturating effect at high values, indicating a weaker association at high richness levels (Fig. 4c), while bacterial and fungal richness themselves were positively and linearly associated (Fig. S5). Biomes such as deserts and montane grasslands exhibited positive richness associations across all three groups (Fig. S6). Strong associations were observed between the richness within fungal guilds and plant richness across biomes (Fig. S7), while associations between the richness within fungal guilds and bacterial richness were weaker. The strongest association was observed between arbuscular mycorrhizal fungi and bacteria in tropical forest biomes (Fig. S8).

**Figure 4:**
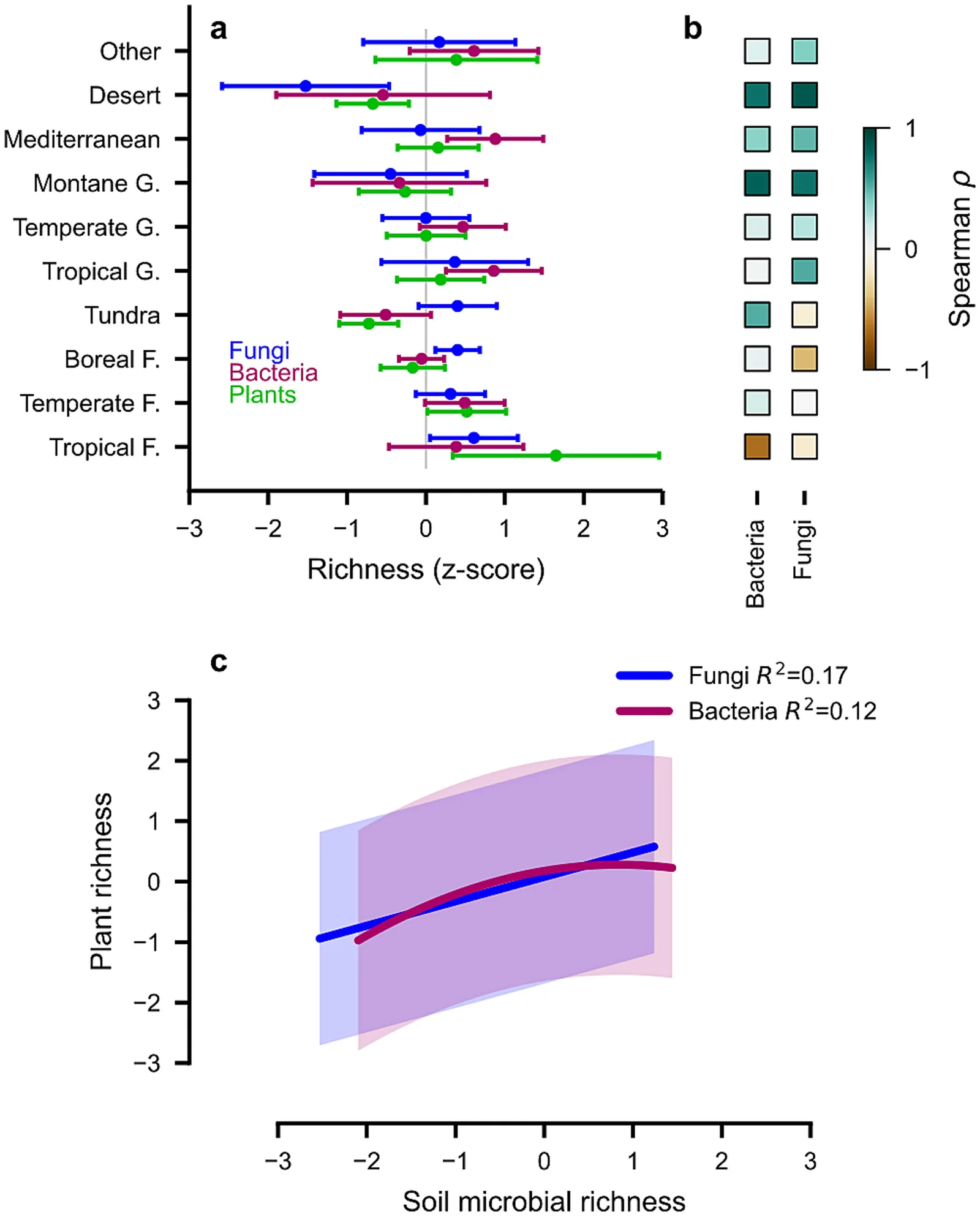
Biome richness and cross-kingdom associations. (a) Standardized area-weighted richness of plants, bacteria, and fungi modeled for different biomes. (b) Biome-specific Spearman correlation of bacteria and plants and fungi and plants. (c) Global links between soil microbial richness and plant richness.

Although overall fungal richness did not exhibit distinct environmental drivers, individual fungal guilds displayed clear biogeographical patterns (Fig. 5). The distribution of foliar and root endophytes and ectomycorrhizal fungi followed the vegetated regions, while saprotrophs had the highest richness in northern latitudes (Fig. 5a). In contrast, arbuscular mycorrhizal fungi showed peak richness at high latitudes (>60°N), in Central Asia and Western China (Fig. 5a). When using the mapped soil bacterial and fungal richness as predictors, they explained virtually none of the variation in plant richness (*R*^2^ = −7 ± 10%, max. *R*^2^ = 13%, *n* = 829). The explanatory power increased slightly when the richness of individual fungal guilds was considered (*R*^2^ = 9 ± 10%, max. *R*^2^ = 28%, *n* = 829). Among the fungal guilds, endophyte richness was the most important predictor of plant richness, with bacterial richness, parasites/pathogens, and saprotrophs also contributing moderately (Fig. 5b). However, only endophyte and ectomycorrhizal fungal richness consistently showed high positive correlations with plant richness while other guilds of comparable size did not (Fig. 5c). For example, the richness of arbuscular mycorrhizal fungal richness was negatively correlated with plant richness, and saprotroph richness showed no correlation (Fig. 5c). The contrasting latitudinal patterns among fungal guilds help explain the lack of consistent biogeographical patterns for overall fungal richness (Fig. 5d). Nonetheless, the richness of bacteria and different fungal guilds showed limited association with the global biogeography of plant richness.

**Figure 5:**
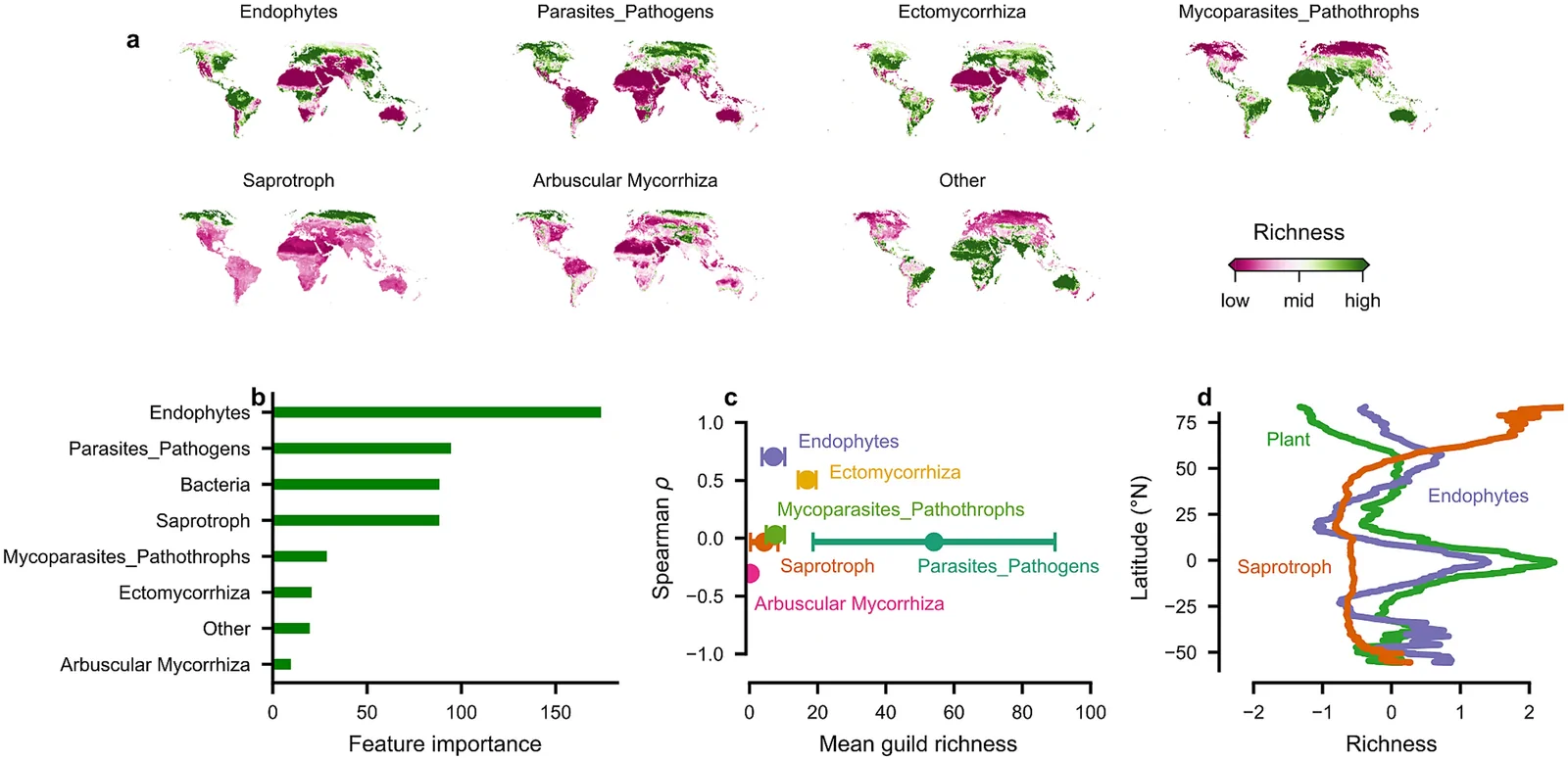
Plant richness links to fungal endophyte richness. (a) Modeled global maps of fungal guild richness. (b) Feature importance for explaining plant richness using bacterial richness and fungal guild richness. (c) Global correlation of fungal guild richness and plant richness (mean and SD). (d) Latitudinal richness patterns for plants, endophytic, and saprotrophic fungi.

## Discussion

We mapped the global patterns of plant, bacterial, and fungal richness and investigated their environmental drivers and hypothesized that soil microbial diversity is linked to plant diversity. Our results confirm a positive association between soil microbial and plant richness, though bacterial richness shows a saturating pattern at high diversity. Plant richness peaks near the equator, while bacterial richness is highest in semi-arid regions. Fungal richness shows less distinct latitudinal patterns, but fungal endophyte richness correlates strongly with plant richness, highlighting complex plant-microbiome interactions across biomes.

The latitudinal pattern of plant diversity is influenced by several factors, including climate, habitat heterogeneity, and evolutionary history [44]. In the context of plant diversity, tropical forests exhibit a remarkable variety of species due to their stable climate, high levels of solar radiation, and abundant rainfall. Contrastingly, bacterial richness was greatest in semi-arid regions and transition zones, including temperate and tropical grasslands, as well as the Mediterranean coastal regions. This observation aligns with prior research indicating that bacterial richness and diversity are typically greater in semi-arid regions than in areas with higher precipitation [45] where many aqueous (micro-) habitats persist and resources are plentiful [46]. In desert grassland ecosystems, bacterial diversity has been observed to diminish along gradients from grasslands to deserts [47] and is reduced with increasing aridity [48]. Transitional zones, such as temperate and tropical grasslands (savannas), display a combination of environmental conditions conducive to maintaining bacterial diversity. These zones frequently function as ecological buffers, sustaining a mosaic of habitats that enhance species richness and are threatened by climate and land use changes. The identified patterns of bacterial richness indicate that community diversity is affected by both primary productivity and soil edaphic characteristics, including pH and bulk density. Soil pH reflects soil mineral weathering and varies with the climatic soil water balance [49]. Thus, it is a useful proxy variable for describing the biogeography of bacterial community structure and diversity in terrestrial ecosystems [50].

For bacteria, soil edaphic factors play an important role in structuring soil microbial communities [17, 46, 48, 50]. Contrary to our expectations, soil texture makes a minimal contribution to bacterial richness, which may indicate that other abiotic factors may exert stronger selective pressures. Gross primary productivity was a dominant driver for plant and bacterial richness, likely reflecting the dependence of these groups on energy and carbon fluxes in terrestrial ecosystems. Fungal richness, on the other hand, is strongly influenced by radiation, a factor often overlooked in microbial ecology, although relations with temperature are reported [42]. Because radiation is also linked to primary productivity, these variables may be collinear, potentially confounding the interpretation of their individual effects. Rainfall frequency significantly affected plant and bacterial richness but had little impact on overall fungal richness. The positive, hump-shaped relationship between bacterial richness and plant richness reflects the complex interplay of biotic and abiotic factors that shape community composition. Although bacterial and fungal richness were positively associated overall, the weak or absent correlations between bacterial richness and plant richness in tropical forests suggest that resource competition or niche partitioning may decouple these relationships in certain biomes. This underscores the need to investigate microbe-plant interactions across spatial and temporal scales.

In contrast to plants and bacteria, fungal diversity displays less pronounced biogeographical variation across the terrestrial biomes despite the role of fungi for plant diversity and ecosystem multifunctionality [14]. Regions of high fungal diversity are identified in areas including central Africa and northern latitudes. The absence of clear latitudinal gradients [42] highlights the unique ecological functions of fungi, which may not correspond directly with the environmental factors affecting plant and bacterial diversity. Fungal diversity seems to be influenced more by functional roles and particular interactions with their environment than by extensive abiotic factors. Our findings reinforce the notion that environmental drivers of diversity vary significantly across functional groups. Analysis at the guild-level reveals that fungi with different lifestyles display contrasting biogeographical distributions, suggesting that functional roles rather than environmental factors mainly drive fungal diversity and their associations with plant productivity [51, 52]. One of the most striking results was the association between soil fungi identified as foliar and root endophytes and plant richness. Endophytes are known to mediate stress tolerance, nutrient acquisition, and pathogen resistance [16, 53]. Their association with plant richness suggests that functional specialization within the fungal microbiome may be related to plant-microbial interactions. The weaker associations between fungal guild richness and bacterial richness also highlight the distinct ecological niches occupied by these groups and the limited overlap in their environmental responses.

Overall, our findings are in line with the DBD hypothesis and the previously observed pattern of stronger associations in low-diversity biomes that saturate in high-diversity biomes for bacteria [24, 25]. For fungi, a positive association with plant richness is observed particularly for the richness of endophytic guilds, although the strength of the association is not dependent on the magnitude of the guilds’ richness. We initially expected that smaller guild sizes would correspond to higher correlations with plant richness due to strong selection for specific taxa. Interestingly, the richness of arbuscular mycorrhizal fungi is not positively associated with plant richness, suggesting niches are filled and EC shapes the diversity associations. Such negative correlations have been previously reported, in particular, between the diversity of arbuscular mycorrhizal fungi and diversity of non-mycorrhizal plants, while positive associations were observed for ectomycorrhizal fungi [54].

This study presents several limitations that warrant consideration. First, the predictive models for fungal richness explained a relatively low proportion of variance, even when improved through guild-level decomposition, suggesting that key environmental or biotic drivers of fungal diversity remain unaccounted for. Second, the use of microbial richness as a predictor of plant richness yielded little explanatory power, indicating that the observed cross-kingdom associations, while statistically detectable, may not reflect strong linkages at the global scale, and local processes may play an important role. Third, potential confounding effects due to collinearity among environmental predictors, such as radiation and gross primary productivity, limit causal inference regarding the relative importance of individual drivers. Fourth, the reliance on amplicon-based richness metrics for microbial diversity and broad functional guild classifications may obscure finer-scale ecological interactions that underpin plant-microbiome associations. Nevertheless, our maps offer a global baseline for understanding cross-kingdom diversity patterns and highlight promising directions (particularly the role of fungal endophytes) for future mechanistic investigations into plant-microbiome interactions across biomes.

## Conclusion

Our study provides a comprehensive framework for understanding the biogeographical patterns of plant, bacterial, and fungal richness and their environmental drivers. By integrating machine learning approaches and guild-level analyses, we offer a novel perspective on the relationships between soil microbiomes and plant diversity at the scale of terrestrial biomes. These findings have significant implications for biodiversity conservation and ecosystem management, particularly in the face of climate change. Understanding the environmental drivers and functional roles of microbial diversity can inform strategies to maintain ecosystem stability and productivity while preserving the diversity of the three major kingdoms. However, our study also highlights important gaps that warrant further investigation. The low predictive power of fungal richness models (despite the highest numbers of samples) underscores the need for more detailed datasets that capture fungal diversity at finer taxonomic and functional resolutions. Additionally, the contrasting responses of fungal guilds to environmental variables suggest that future studies should prioritize guild-specific analyses to unravel the ecological roles of fungi in different biomes. Furthermore, we note that the African continent as well as large regions in Asia and central Australia are not well represented in global datasets, even though they seem to harbor high microbial diversity. Nonetheless, the use of microbial functional guild richness may provide an avenue for exploring the functional links between soil microbiomes and plant communities. While we were able to explain a small portion of plant richness variation using fungal guilds, further research is needed to assess how microbial biomass and specific functional traits, rather than richness alone, influence plant diversity. Integrating these functional dimensions [14, 42, 55] into biogeographical studies will be crucial for advancing our understanding of terrestrial ecosystem dynamics.

## Supplementary Material

2026_-_09_-_10_plant_and_soil_microbial_diversity_R2_SI_ycag266

## Data Availability

The data used in this work were obtained from the following DOIs: 10.5281/zenodo.3366252, 10.15156/BIO/2263453, 10.6084/m9.figshare.19539085, for bacterial, fungal, and plant richness, respectively. The maps generated during the current study are available on Zenodo: 10.5281/zenodo.21133869.
