# Supplementary material for: Associations among soil microbial and plant richness across global terrestrial biomes
Source: ISME Commun. 2026 Sep 18;6(1):ycag266. doi: 10.1093/ismeco/ycag266 (PMC13637820; doi:10.1093/ismeco/ycag266)
Supplement: Supplementary file 1 [file 2026_-_09_-_10_plant_and_soil_microbial_diversity_r2_si_ycag266.pdf]

Supplementary Information for:

Associations among soil microbial and plant richness across global terrestrial biomes

Samuel Bickel[1*], Ahmed Abdelfattah[2], Ayco J. M. Tack[3], Wisnu Adi Wicaksono[1], and Gabriele Berg[1,2,4*]

Affiliation

[1]Institute of Environmental Biotechnology, Graz University of Technology, Graz, Austria

[2]Leibniz Institute for Agricultural Engineering and Bioeconomy (ATB), Potsdam, Germany

[3]Department of Ecology, Environment and Plant Sciences, Stockholm University, SE-106 91 Stockholm, Sweden

[4]Institute for Biochemistry and Biology, University of Potsdam, Potsdam, Germany

*Corresponding authors: samuel.bickel@tugraz.at, gabriele.berg@tugraz.at

Supplementary Figures

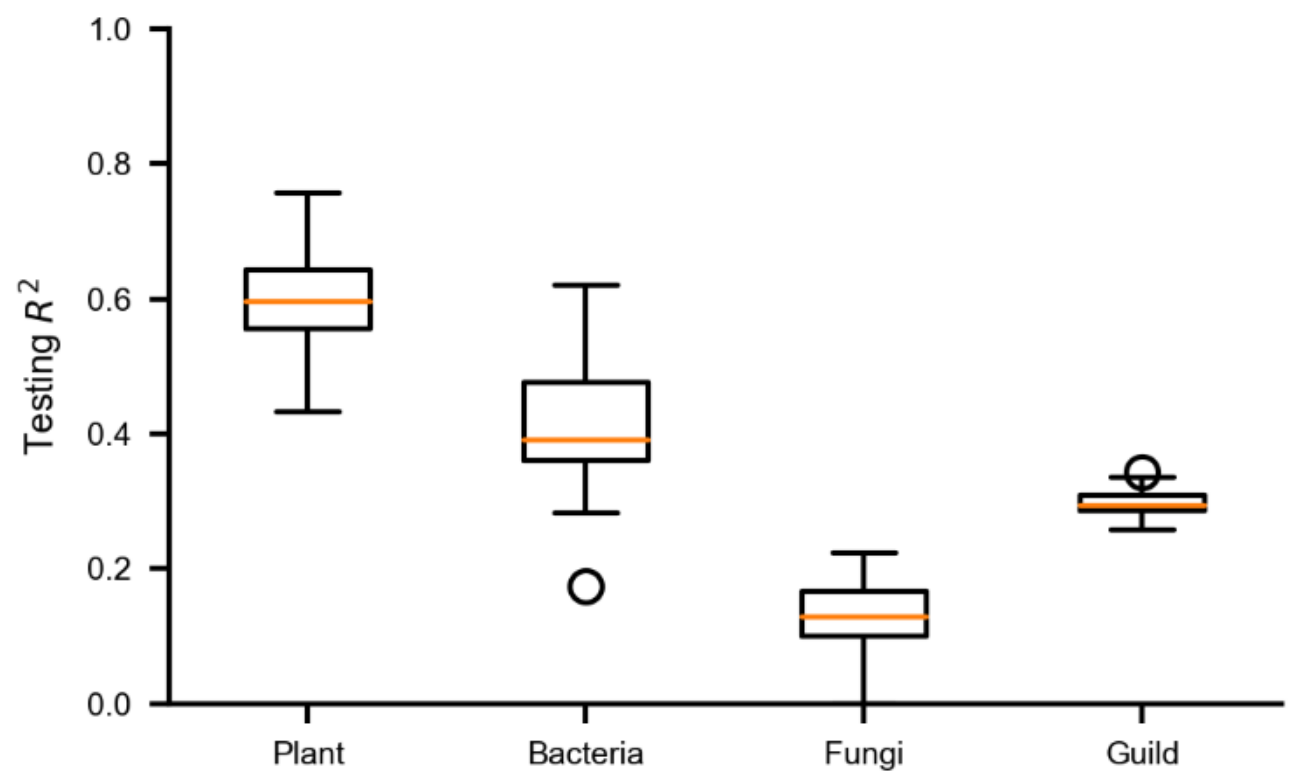

SI Figure 1: Model performance for plant and soil microbial richness. Four models to predict plant, bacterial and fungal and fungal guild richness. R^2 of the testing set.

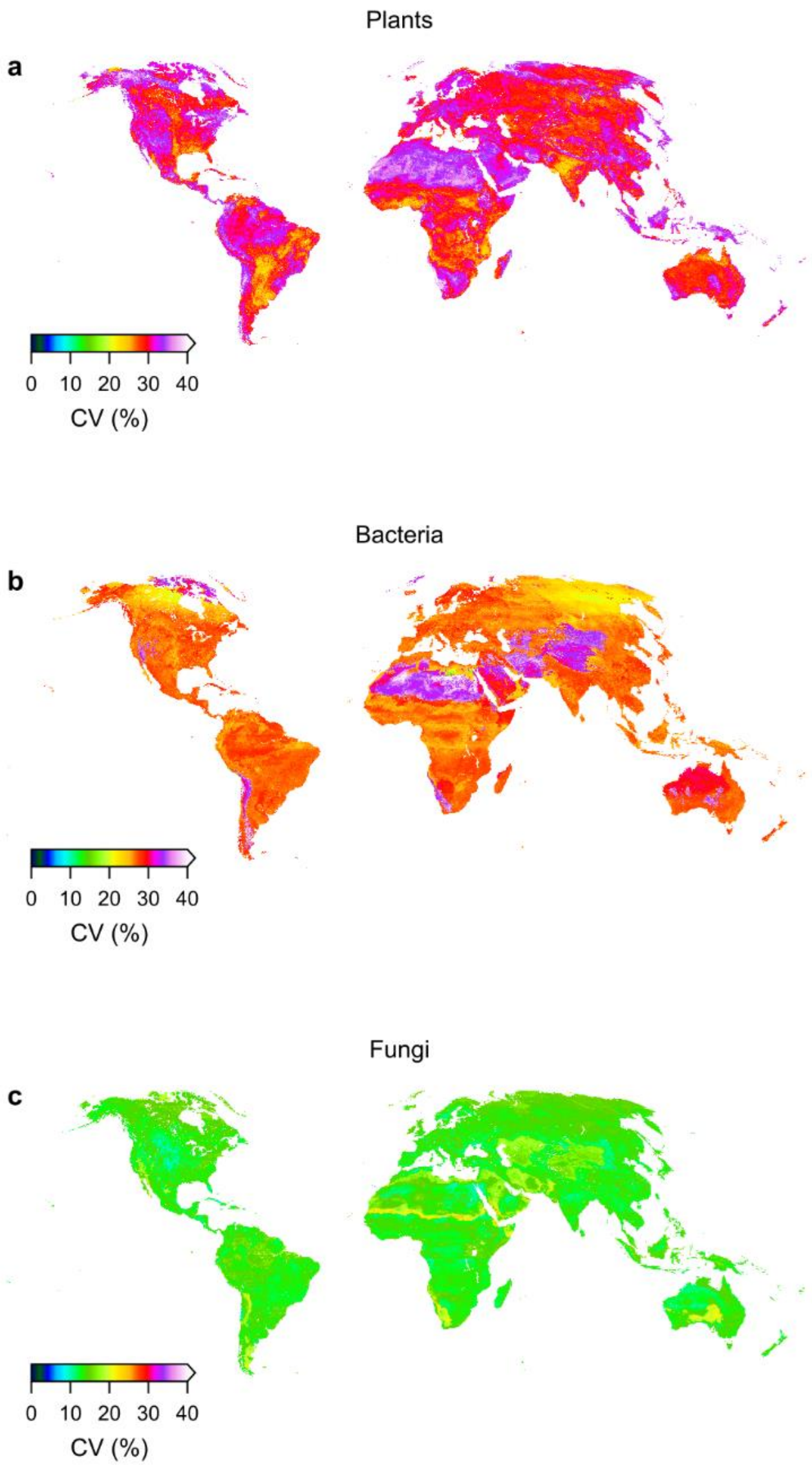

SI Figure 2: Model uncertainty for plant and soil microbial richness. An ensemble of 50 models was averaged to compute the coefficient of variation (standard deviation/mean x 100%).

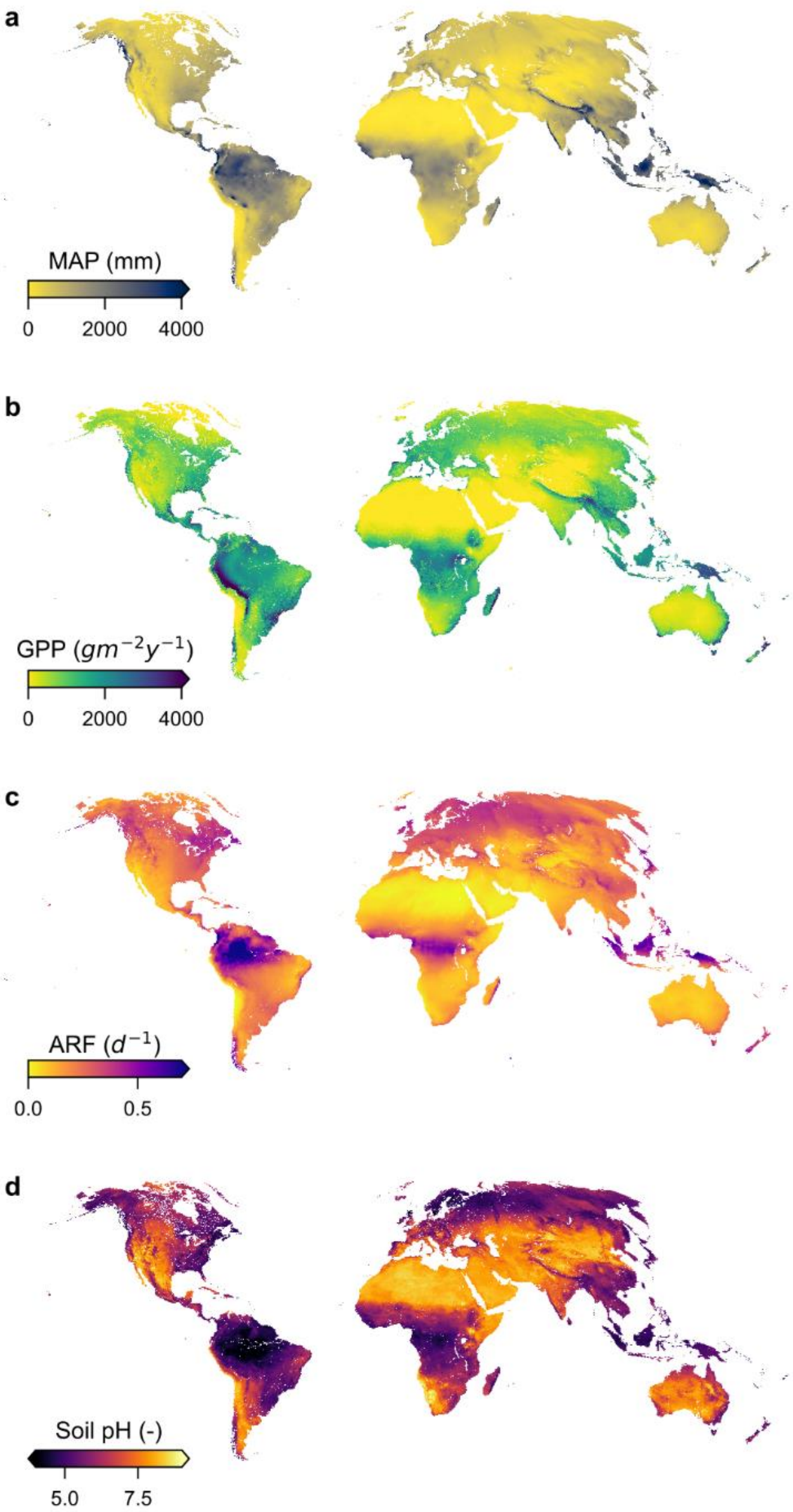

SI Figure 3: Spatial distributions of environmental drivers with diverging patterns among plant, bacterial and fungal richness. a, mean annual precipitation (MAP), **b**, gross primary productivity (GPP), **c**, average rainfall frequency (ARF), and **d**, soil pH in water.

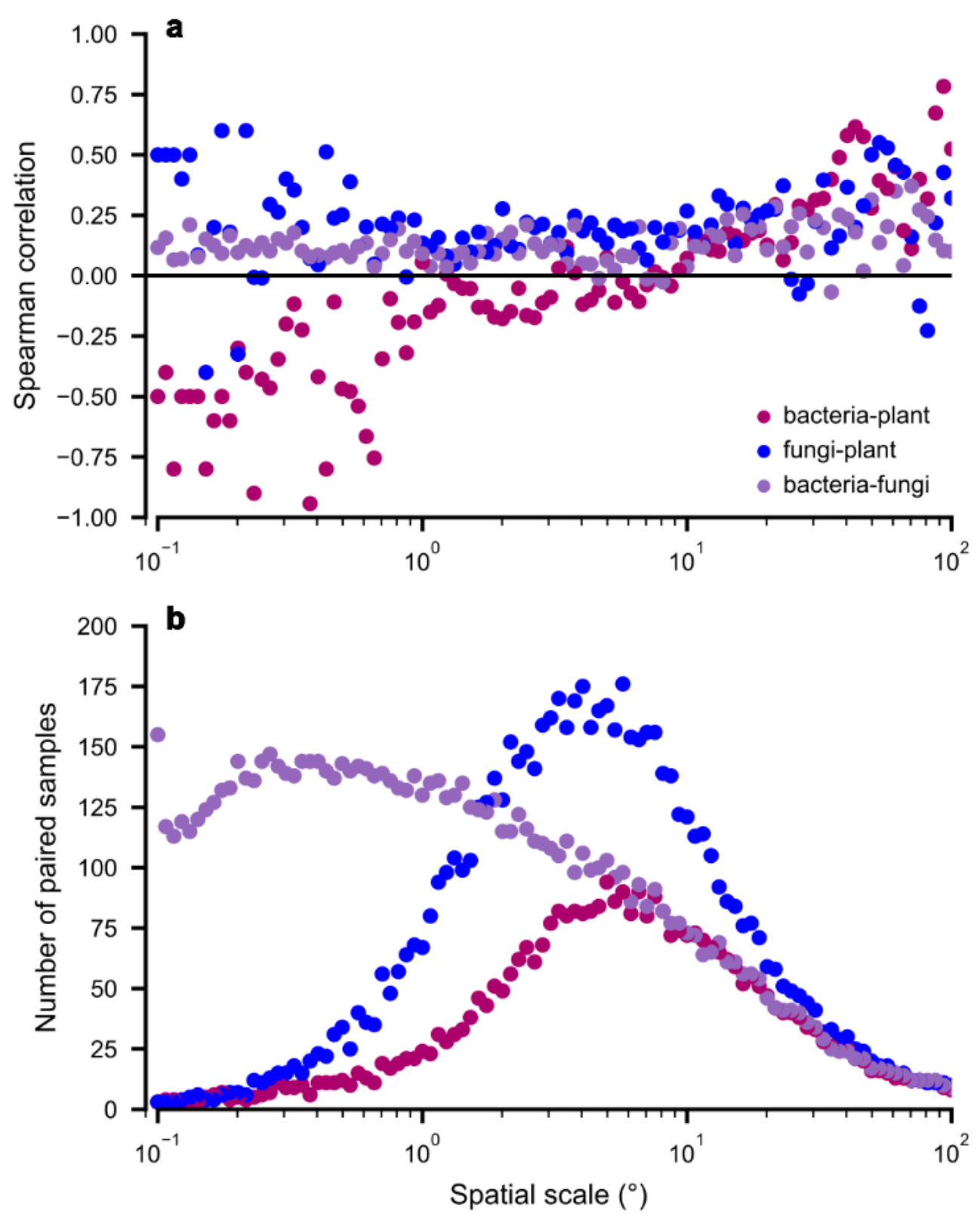

SI Figure 4: Spatial coarse graining of sample locations. a, plant-microbe and microbe-microbe associations, based on **b,** sample pairs obtained from aggregating sample locations at different spatial scales (0.1° to 100°).

SI: Microbe-plant richness across biomes

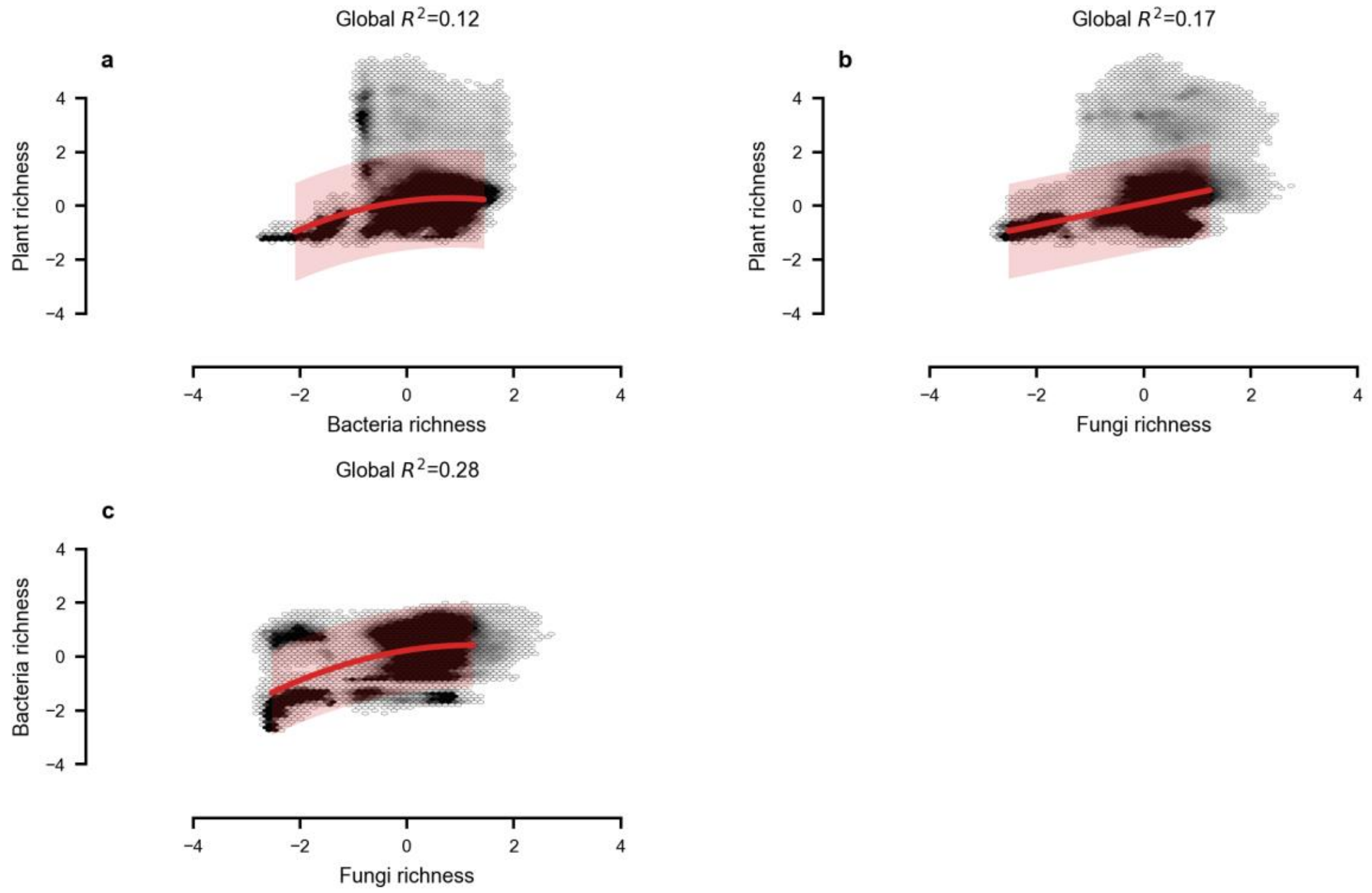

SI Figure 5: Global diversity association for plant and soil microbial richness. Soil diversity association using the standardized potential richness based on the predicted global maps for **a**, plant-bacteria, **b**, fungi-plant, and **c**, fungi-bacteria richness.

SI: Microbe-plant richness across biomes

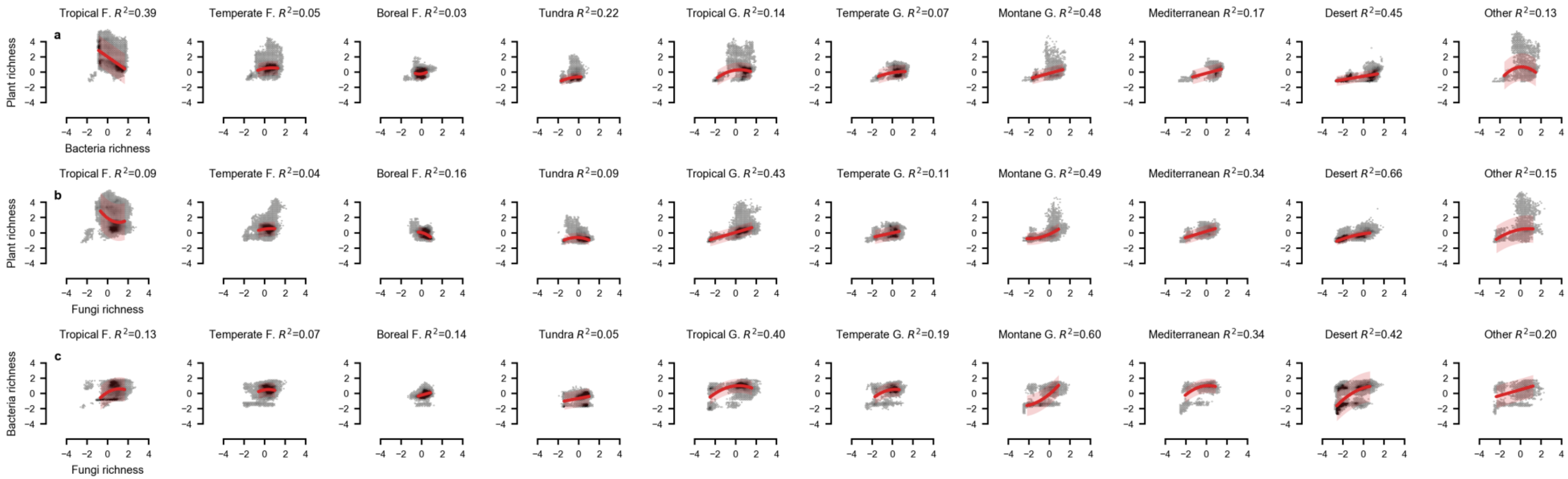

SI Figure 6: Biome-specific diversity association for plant and soil microbial richness. Soil diversity was associated with standardized richness based on predicted global maps. **a**, plant-bacteria, **b**, fungi-plant, and **c**, fungi-bacteria richness. The distributions of modeled values are shown.

SI: Microbe-plant richness across biomes

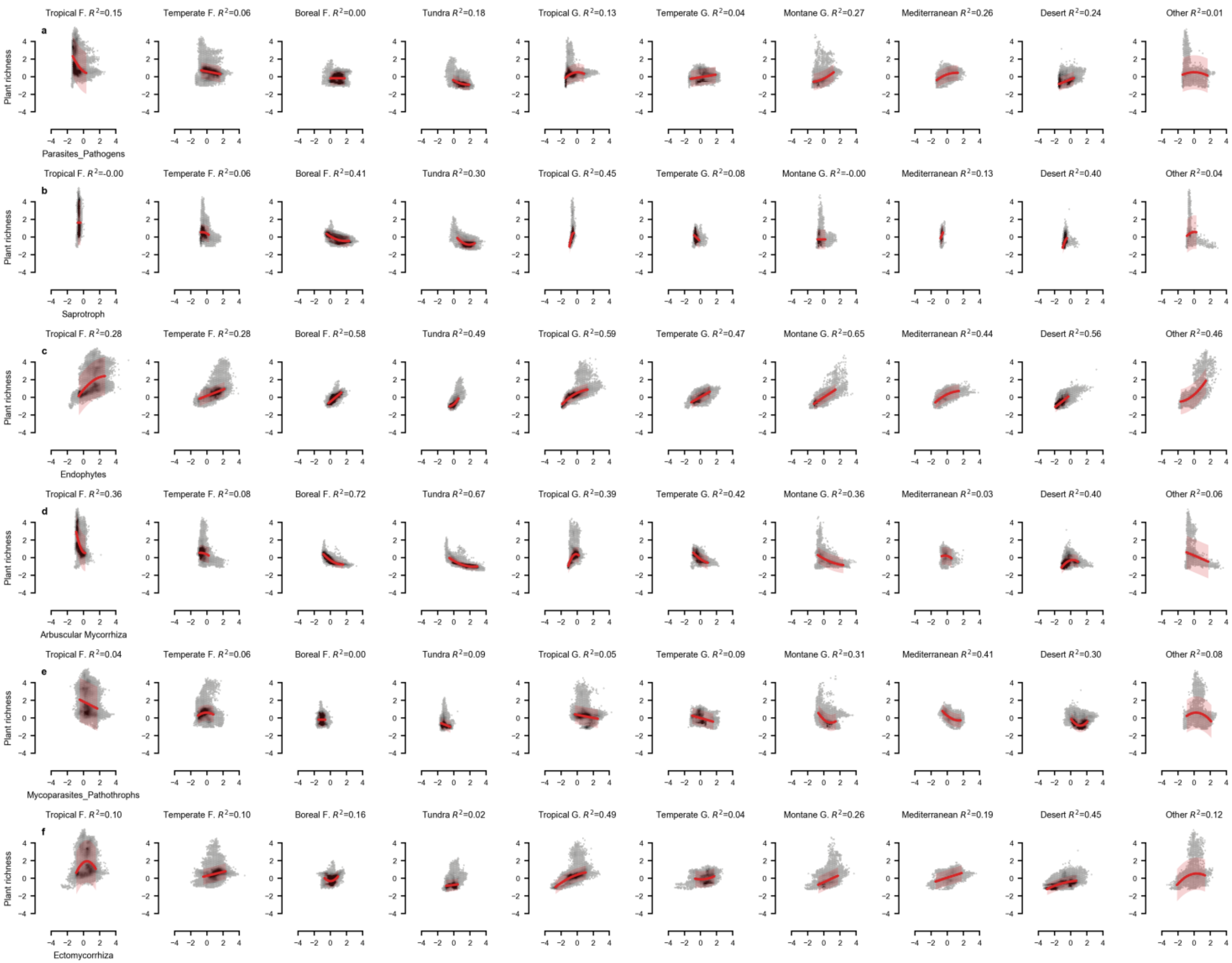

SI Figure 7: Biome-specific association for plant richness and fungal guild richness. a-f, standardized potential richness based on the predicted global maps for each fungal guild. The distributions of modeled values are shown.

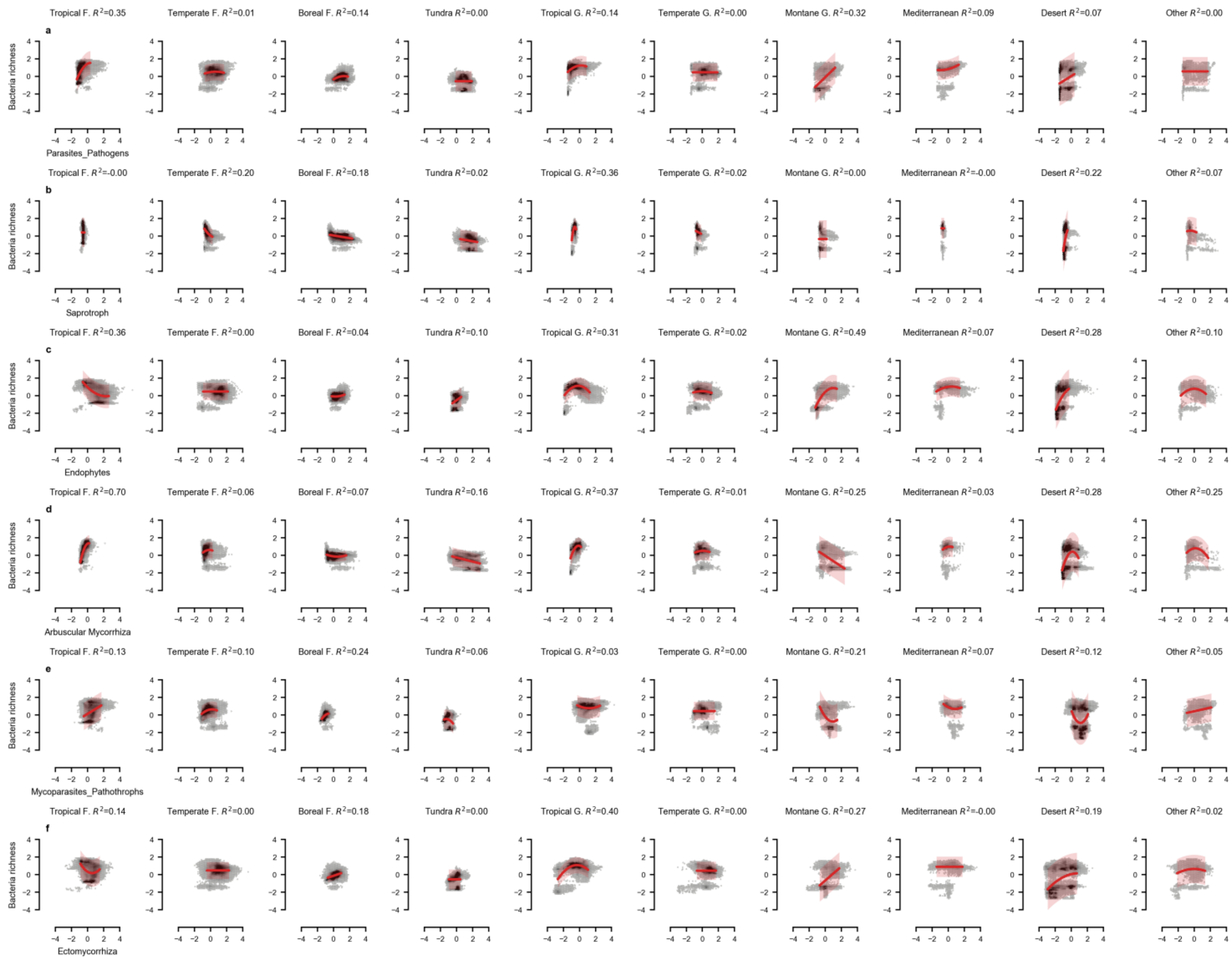

SI Figure 8: Biome-specific association for bacterial richness and fungal guild richness. a-f, standardized potential richness based on the predicted global maps for each fungal guild. The distributions of modeled values are shown.

SI Table 1: Summary of variables used for biogeographical models.

Type	Name	Abbreviation	Units	Source
**Spatial**	Area of sampling grid cell	ARE	km^2	-
**Climate**	Average rainfall frequency	ARF	d^{-1}	[1, 2]
	Mean annual temperature	MAT	°C	[3]
	Radiation	RAD	$W\ m^{-2}$	[3]
	Mean annual precipitation	MAP	mm	[1]
**Vegetation**	Gross primary productivity	GPP	$g\ m^{-2}\ y^{-1}$	[4]
**Soil**	Bulk density	BLD	$g\ cm^{-3}$	[5]
	Soil pH in H_2O	PHH	-	[5]
	Soil type diversity	STD	Number of soil types	[5]
	Texture	TXT	log(%Sand/(%Clay+%Silt))	[5]

SI Table 2: Aggregation of fungal guilds [6].

Guild	Original grouping
Arbuscular Mycorrhiza	arbuscular_mycorrhizal
Ectomycorrhiza	ectomycorrhizal
Endophytes	foliar_endophyte
Endophytes	root_endophyte
Mycoparasites/Pathothrophs	mycoparasite
Mycoparasites/Pathothrophs	unspecified_pathotroph
Other	animal_endosymbiont
Other	animal-associated
Other	arthropod-associated
Other	epiphyte
Other	lichenized
Other	moss_symbiont
Other	protistan_parasite
Other	sooty_mold
Other	unspecified
Other	unspecified_symbiotroph
Parasites/Pathogens	algal_parasite
Parasites/Pathogens	animal_parasite
Parasites/Pathogens	lichen_parasite
Parasites/Pathogens	plant_pathogen
Saprotroph	dung_saprotroph
Saprotroph	litter_saprotroph
Saprotroph	nectar/tap_saprotroph
Saprotroph	pollen_saprotroph
Saprotroph	soil_saprotroph
Saprotroph	unspecified_saprotroph
Saprotroph	wood_saprotroph

References

1. Beck HE, Wood EF, Pan M, Fisher CK, Miralles DG, van Dijk AIJM, et al. MSWEP V2 Global 3-hourly 0.1° precipitation: methodology and quantitative assessment. *Bull Am Meteorol Soc* 2019;**100**:473–500. https://doi.org/10.1175/BAMS-D-17-0138.1
2. Bickel S, Or D. Dataset of global climatic soil water contents and consecutive dry days. 2021. Zenodo, 2021.
3. Fick SE, Hijmans RJ. WorldClim 2: new 1-km spatial resolution climate surfaces for global land areas: new climate surfaces for global land areas. *Int J Climatol* 2017;**37**:4302–4315. https://doi.org/10.1002/joc.5086
4. Qiu R, Han G, Ma X, Xu H, Shi T, Zhang M. A Comparison of OCO-2 SIF, MODIS GPP, and GOSIF Data from Gross Primary Production (GPP) Estimation and Seasonal Cycles in North America. *Remote Sens* 2020;**12**:258. https://doi.org/10.3390/rs12020258
5. Hengl T, Jesus JM de, Heuvelink GBM, Gonzalez MR, Kilibarda M, Blagotić A, et al. SoilGrids250m: Global gridded soil information based on machine learning. *PLOS ONE* 2017;**12**:e0169748. https://doi.org/10.1371/journal.pone.0169748
6. Tedersoo L, Mikryukov V, Anslan S, Bahram M, Khalid AN, Corrales A, et al. The Global Soil Mycobiome consortium dataset for boosting fungal diversity research. *Fungal Divers* 2021;**111**:573–588. https://doi.org/10.1007/s13225-021-00493-7
